# LiveWell in early childhood: results from a two-year pilot intervention to improve nutrition and physical activity policies, systems and environments among early childhood education programs in South Carolina

**DOI:** 10.1186/s12889-021-10975-7

**Published:** 2021-05-13

**Authors:** Meghan Slining, Sally Wills, Melissa Fair, Jen Stephenson, Stephanie Knobel, Misty Pearson, Tia Prostko, Joanna Smyers, Joanne Timberlake, Miguel Negrete

**Affiliations:** 1 Health Sciences Department, Furman University, 3300 Poinsett Highway, Greenville, SC USA; 2 LiveWell Greenville, Greenville, SC USA; 3 Institute for the Advancement of Community Health, Furman University, Greenville, SC USA; 4 YMCA Judson Community Center, Greenville, SC USA; 5 SC Department of Health and Environmental Control, Division of Nutrition, Physical Activity, and Obesity Prevention, Columbia, SC USA; 6 Early Care and Education Division, South Carolina Department of Social Services, Greenville, SC USA; 7 Youth Learning Institute, Clemson University, Clemson, SC USA

**Keywords:** Nutrition, Physical activity, Child care, Overweight, Policy, Public health

## Abstract

**Background:**

Early childhood education (ECE) settings are critical intervention targets for obesity prevention. This study evaluated a pilot two-year community-based participatory research (CBPR) project designed to assist ECE center directors and caregivers in policy, systems and environmental (PSE) change for improving healthy eating (HE) and physical activity (PA).

**Methods:**

A two-year CBPR study was conducted in 10 licensed ECE centers in Greenville, South Carolina. The intervention consisted of five steps: [1] baseline data collection and self-assessment using the Nutrition and Physical Activity Self-Assessment for Child Care (Go-NAP SACC), [2] tailored goal setting and action planning, [3] technical assistance and access to resources, [4] post intervention data collection and re-assessment, and [5] celebration of success. Main outcome measures (HE and PA environments, practices and policies) were assessed using the Environment and Policy Assessment and Observation (EPAO) tool at baseline and 24 months. One classroom of 3–5-year-olds was randomly selected for observation from each center (mean of 12 children per classroom). Means and standard deviations were calculated for total PA, total nutrition and each subscale of PA and nutrition. Paired sample t-tests were calculated to assess changes in EPAO scales from baseline to post intervention.

**Results:**

Ten ECE centers enrolled in the pilot study and eight completed the two-year intervention. Center-based goals were accomplished across all 8 ECE centers over the two-year intervention: 16 child nutrition goals, 6 outdoor play goals, 11 physical activity goals and 8 screen time goals across the entire sample. Nutrition policy and PA policy significantly improved (*p* < 0.05), with greater improvements in PA (10.0 point increase, *p* = .048) as compared to nutrition (3.3 point increase, *p* = 0.02).

**Conclusions:**

Utilizing a CBPR approach, this two-year nutrition and PA PSE intervention in ECE centers improved ECE center HE and PA policies.

## Background

Twenty eight percent of South Carolina (SC) preschoolers had overweight in 2010 [1], and 31.5% of Greenville County, SC Head Start children aged two to five had overweight or obesity in 2015 [2]. Evidence demonstrates immediate and long-term risks associated with overweight and obesity during childhood [3, 4]. Poor diets and lack of physical activity (PA) increase children’s risk for overweight and related health problems [5]. Early childhood has been acknowledged as a critical time for the development of eating and activity patterns [6, 7]. Further, both sedentary behaviors and PA appear to track consistently from early childhood to middle childhood [7].

Childcare settings are critical intervention targets. In 2016, 60% of children age five and younger were in a nonparental care arrangement, with 59% of those attending center-based care [8]. Children in center-based care spent an average of 24 h per week in ECE settings [8], with some receiving the majority of their daily calories in those settings [9]. While children in childcare often do not meet national dietary, activity or sedentary behavior guidelines [10–12], these settings have been shown to be important predictors of behavior [13].

Childcare environmental and policy interventions have been identified as important population-based approaches to childhood obesity prevention [14, 15]. While the Health and Medicine Division (formerly known as the Institute of Medicine (IOM)) has provided policy recommendations designed to prevent early childhood obesity by promoting healthy childcare environments [14], few states have implemented regulations in line with their recommendations [16, 17]. Furthermore, research in SC suggests that implementation of regulations or standards alone results in only modest changes in PA practices [18]. Importantly, a variety of strategies (including environmental self-assessment, tailored goal setting, educational workshops for childcare staff and parents, technical assistance, and access to resources) have been shown to have a positive impact on the implementation of healthy eating and active living policies in the ECE setting [19–27]. Technical Assistance (TA) is a particularly promising strategy to bridge recommendations and practice. Providing direct support to ECE directors, TA builds capacity for implementation of recommendations [28] and narrows the gap between science and practice [29]. Several nutrition and PA intervention studies in ECE settings have successfully utilized TA as an implementation strategy [30], suggesting it may be necessary for ECE interventions to be successful [31, 32].

Community-based participatory research (CBPR) is “a collaborative approach to research that equitably involves all partners in the research process and recognizes the unique strengths that each brings.” [33] Participatory research models, whereby academic and community partners are actively engaged throughout the research process, have been recognized as key to the national prevention research agenda [34]. Few studies in the ECE setting have employed these methods. A recent review characterizing the process and dynamics of community-based childhood obesity prevention interventions identified only three of 13 studies utilizing a CBPR approach, with none of those conducted in children under 5 years of age [35].

LiveWell Greenville (LWG) is a community coalition of over 200 non-profit, government, and corporate partners working to promote policy, system, and environmental (PSE) change to enhance healthy eating and active living opportunities for Greenville County residents. LWG was asked to establish an initiative to provide TA support to create PSE change in ECE centers in Greenville County. Utilizing a community-based participatory research (CBPR) approach, LWG launched a two-year ECE intervention in 10 Greenville County childcare centers in August of 2016. The purpose of this study was to determine the impact of the two-year intervention on childcare center food and PA policies, practices and environments.

## Methods

The LWG Early Childhood Initiative was developed in direct response to demand from local ECE stakeholders: two ECE center directors, the president of Greenville County Childcare Association, and staff from an early childhood development non-profit. Over a period of several months, LWG acted as an intermediary, facilitator, and investigator by furthering conversations with stakeholders to determine gaps in Greenville County’s existing ECE system. Following initial conversations among state and local ECE stakeholders with a focus on obesity prevention, the ECE Community Advisory Committee (ECCAC) was formed consisting of 11 stakeholders from local ECE centers, ECE development non-profits, governmental agencies and the local university.

The first task of the ECCAC was to develop a community action plan. LWG hired a part-time consultant to facilitate the action plan development. The consultant was a former LWG volunteer with existing partnership relationships and ability to facilitate the process from a neutral position. In July 2015, the consultant facilitated a strengths, weaknesses, opportunities and threats (SWOT) analysis in which partners individually identified parameters to improving HE and PA in ECE settings in Greenville County. The consultant then facilitated a discussion in which the partners prioritized each list and discussed the following questions: How can we maximize the use of our collective strengths? How can we overcome threats and weaknesses? How can we take advantage of our opportunities? Following the SWOT analysis, from August 2015–February 2016, the ECCAC met monthly to establish goals and determine strategies to achieve their goals. At each meeting, the consultant worked to build trust so that partners could engage in difficult conversations to identify common goals. The consultant ensured transparency at every meeting and regularly communicated with partners between monthly meetings.

In accordance with CBPR principles, the ECCAC collaboratively developed an intervention, study methods and dissemination of results.

To ensure a commitment to a CBPR approach, the evaluator and the LWG partnership coordinator participated in the Community Engaged Scholars Program through the South Carolina Clinical and Translational Research Institute at the Medical University of South Carolina. The CES-P is a 9-month training program designed to increase capacity of community-academic partnerships to conduct community-based health research with mutual ownership of the process and products.

### Study design and participants

The ECCAC conducted a one-group pre-test/post-test evaluation of a pilot intervention designed to assist ECE center directors and caregivers to improve nutrition and PA policies and practices. This two-year intervention took place from August of 2016 through August of 2018 in 10 licensed ECE centers located in Greenville, SC. ECE centers were recruited from a list of centers participating in Palmetto Shared Services [36], a pilot project designed to help SC childcare providers save time and money through shared purchasing power and access to online resources. ECE centers who were already participating in existing technical assistance interventions identified in the state were excluded. Our resulting sample contained a higher proportion of care provided by faith-based organizations (60%) as compared to both Greenville County (27%) and the national average of of center care provided by faith-based organizations (9%) [37]. An LWG experienced health educator called or visited each center to invite them to participate in the study. Center directors provided written consent prior to the baseline data collection. Because identifying information was not collected on the children receiving care, parental consent was not collected. All study methods were approved by the Furman University Institutional Review Board.

### Intervention

The LWG ECE intervention was designed to assist ECE center directors and caregivers to improve healthy eating and active living policies and practices in ECE centers. Recognizing that behavior is affected by multiple levels of influence and that effective interventions target changes in multiple domains, the intervention utilizes the socioecological framework [38]. Early in the intervention development process, the evaluator presented to the ECCAC on the socioecological framework and its use in obesity prevention interventions. The ECCAC then used this information as they designed the LWG ECE intervention. Specifically, the ECCAC intended to facilitate individual level changes in child HE and PA behaviors (intrapersonal domain) through the intentional creation of a network of childcare center directors and caregivers (interpersonal domain) and through education and technical assistance on appropriate changes in HE and PA policies, systems and environments (environmental domain).

The LWG ECE Initiative consisted of five steps: (1) baseline EPAO data collection and self-assessment using the Nutrition and Physical Activity Self-Assessment for Child Care (Go NAP SACC), (2) tailored goal setting and action planning, (3) group- and individual-level technical assistance, networking and access to curated resources, (4) post EPAO data collection, and (5) celebration of success.

Participating centers were provided access to the Go NAP SACC and online resources for goal setting and achievement. Center directors and caregivers completed four modules of the online Go NAP SACC self-assessment [39]: Child Nutrition, Infant & Child Physical Activity, Outdoor Play & Learning and Screen Time. These assessments allowed providers to self-assess nutrition and PA policies and environments and were completed at three time points: once prior to the intervention, once during the intervention (mid-point survey at approximately 12 months) and again following the intervention (post survey at approximately 24 months).

Participating centers received both individual and group technical assistance (TA) from an experienced LWG health educator, providing expert advice and guidance related to nutrition and PA. Specifically, the LWG health educator helped ECE directors identify resources relevant to their center goals (e.g., examples of parent handbooks or policy language from local centers) Individualized one-on-one TA was provided to ECE directors an average of three times per year. The ECCAC planned for individualized TA to be delivered once per quarter, with the exception of the summer quarter as many centers were closed. Following self-assessment and goal setting, the TA guided participants in developing action plans and provided regular, ongoing support for achievement of goals. Group TA was provided in-person to the cohort of participating centers through eight networking sessions (four per year) where all centers jointly received TA on making improvements toward their goals. The topics of each networking session were determined based on commonly shared goals among centers as well as feedback solicited from centers. Networking sessions included substantial emphasis on accountability and developing social support amongst childcare center directors and caregivers. As incentives, participants received lunch and a variety of small items for the center at each session. Items included portable play equipment, music CD’s, and gardening materials.

### Outcome measure

Childcare center food and PA policies, practices and environments were assessed using the Environment and Policy Assessment and Observation (EPAO) instrument designed to assess childcare environments [40–43]. The 2005 version of the EPAO consists of 75 items that, when scored, result in scores for total nutrition and total PA as well as 16 subdomains (1) nutrition policy, 2) nutrition training and education, 3) staff nutrition behaviors, 4) nutrition environment, 5) fruits and vegetables provided, 6) grains, beans and lean meats provided, 7) high sugar, salt and fat foods provided, 8) beverages, 9) PA policy, 10) PA training and education, 11) staff PA behaviors, 12) active opportunities provided, 13) sedentary opportunities provided, 14) sedentary environment, 15) portable play equipment, 16) fixed play equipment. Item responses were converted to a three-point scale (zero, one and two), and totaled within each subdomain, for a possible 20 points in each subdomain. Total nutrition and total PA scores were calculated by summing each of the relevant subdomains and dividing by 20.

### Data collection

Data collectors were trained by a study investigator over a two-day period. Training included assessment of all EPAO items and scoring criteria, observational data collection techniques, practice scoring of various scenarios, a mock observation in an ECE center, and data entry and scoring procedures.

The EPAO data was collected in June of 2016 (baseline) and in August of 2018 (post intervention). Teams of two trained data collectors observed one (randomly selected) 3–5-year-old classroom at each ECE center. Data collectors arrived 15 min prior to center opening and left at the end of the ECE day (or 5 pm, whichever came first). During the observation, data collectors observed and scored all program staff and child PA and eating practices and policies. Two data collectors were present to facilitate enhanced data collection (ability to visit kitchen staff with questions, while still observing activities in the classroom). Following data collection, study staff compared scoring and discussed any discrepancies before scoring and entering data. Data collectors were not part of the intervention staff and did not interact with the ECE centers outside of data collection events.

Information on the ECE center was collected from the director of each center by the LWG health educator during phone or in-person interviews following baseline data collection.

### Statistical analysis

Descriptive statistics were calculated for center demographics, total PA, total nutrition and each subscale of PA and nutrition. Paired sample t-tests were calculated to assess changes in EPAO scales from baseline to post intervention. All analyses were performed using Stata 15 (College Park, TX) and differences were considered significant at *p* < .05.

## Results

We enrolled 10 ECE centers and collected baseline data in August of 2016. Over the course of the two-year intervention, one center withdrew (stating time constraints as the reason for withdrawal) and two centers merged, resulting in eight ECE centers that completed post intervention data collection in August of 2018. Baseline characteristics of ECE centers are included in Table 1. For the 10 centers enrolled at baseline, the mean (SD) number of children enrolled was 122.8 (73.7) and the mean (SD) years in operation was 25.5 (15.9). Sixty percent of centers were faith-based organizations and 10% participated in the Child and Adult Care Food Program (CACFP).

**Table 1:** Baseline demographic characteristics of ten early childcare education centers in Greenville, South Carolina, USA, 2016–2018

Years in operation (Median, IQR)	23.5 (16–35)
Enrollment (Median, IQR)	125 (76–188)
Children per classroom (Mean, range)	12 (7–16)
CACFP Participation (%)	10
Faith-based (%)	60
Years in operation (Mean, SD)	25.5 (15.9)
Enrollment (Mean, SD)	122.8 (73.7)
CACFP Participation (%)	10
Faith-based (%)	60

There were no significant differences between the ECE center that withdrew from the study and those retained in terms of baseline characteristics, except for years of operation. The ECE that withdrew had fewer years in operation (2 years) than those who completed the study (mean of 25.5 years in operation).

After sharing aggregate baseline EPAO results with ECE center directors, the cohort selected two common cohort goals for the intervention period (teachers and staff receive professional development on 1) PA and 2) nutrition two times per year or more). In addition, each center director selected additional nutrition and PA areas for improvement based on the results of their Go NAP SACC self-assessments (setting two nutrition and two PA goals each year of the intervention). A total of 16 goals across all eight ECE centers were accomplished for child nutrition (average of two goals per center, range 0–6). In addition to the cohort goal of increasing teacher and staff professional development on nutrition, centers accomplished goals such as increasing offerings of dark green, orange, red or deep yellow vegetables, increasing posters, books and other learning materials promoting healthy eating, teachers asking if children are hungry before serving more food, praising and providing hands-on help to toddlers as they learn to feed themselves, incorporating planned nutrition education into classroom routines, talking informally with children about healthy eating and developing written policy on child nutrition. A total of six goals across all eight ECE centers were accomplished for outdoor play (average of 0.75 goals per center, range 0–3). Centers accomplished goals including increasing outdoor playtime provided to children each day, increasing the amount of portable play equipment available for child use outdoors, increasing teacher and staff professional development on outdoor play and learning and increasing family education on outdoor play and learning. A total of 11 goals across all eight ECE centers were accomplished for PA (average of 1.4 goals per center, range 0–2). Centers accomplished goals including increased time for PA each day, limiting seated time for preschool children and toddlers, increasing portable play equipment for indoor use, teachers encouraging and joining in to increase children’s PA, teachers not restricting PA as a means to manage challenging behaviors, teachers and staff receiving professional development on children’s PA and increasing family education on children’s PA. Finally, a total of eight goals across all eight ECE centers were accomplished for screen time (average of 1 goal per center, with range of 0–3). Accomplished goals included offering alternate activities for children when screen time was offered, talking with children about what they were seeing and learning when screen time was offered, teachers and staff receiving professional development on screen time, families receiving education on screen time and developing written policies on screen time.

Table 2 provides center EPAO scores at baseline and at post intervention. Post intervention, total EPAO nutrition and PA scores increased (1.96 point increase in nutrition and 1.24 point increase in PA), though change in total scores was not statistically significant. Among EPAO subscales, ECE centers significantly improved both their nutrition policy scores (from a mean of 9.17 to 13.75 (*p* < 0.05)) and their PA policy scores (from a mean of 10.0 to 17.5 (p < 0.05)). While baseline and post intervention scores were not significantly different for any of the other subscales, a number had moderate effect sizes (total nutrition score *d* = 0.79, physical activity training and education *d* = .78, grains, beans and lean meat provided *d* = 0.77, staff physical activity behaviors *d* = 0.74, and nutrition environment *d* = 0.72).

**Table 2:** EPAO scores pre and post intervention among 8 early childcare centers in Greenville, South Carolina, USA, 2016–2018 ^a^

	Pre-Intervention	Post-Intervention	Difference		Cohen’s *d*
EPAO subscale items	Mean ± SD	Mean ± SD	Mean ± SD	*p* value	
Total nutrition score	9.76 ± 2.47	11.72 ± 2.79	1.96 ± 2.48	0.06	0.79
Nutrition policy	9.17 ± 6.11	13.75 ± 6.77	4.58 ± 4.34	0.02*	1.05
Nutrition training and education	5 ± 2.83	6 ± 4.28	1 ± 4.66	0.56	0.21
Staff nutrition behavior	11.75 ± 5.33	14.16 ± 2.67	2.41 ± 5.18	0.23	0.47
Nutrition environment	8.33 ± 6.9	11.88 ± 4.8	3.54 ± 4.91	0.08	0.72
Fruits and vegetables provided	10.25 ± 3.65	12 ± 3.85	1.75 ± 3.62	0.21	0.48
Grains, beans and lean meat provided	3.92 ± 4.7	8.29 ± 6.45	4.38 ± 5.7	0.07	0.77
High fat/high sugar provided	15.63 ± 3.34	14.63 ± 3.54	1.31 ± 2.09	0.47	0.27
Beverage	12.27 ± 3.79	13.05 ± 4.88	0.77 ± 4.29	0.63	0.18
Total physical activity score	11.76 ± 1.54	13 ± 2.18	1.24 ± 2.34	0.17	0.53
Physical activity policy	10 ± 9.26	17.5 ± 7.07	7.5 ± 8.86	0.048*	0.85
Physical activity training and education	2.81 ± 3.88	10.94 ± 7.43	8.13 ± 10.42	0.06	0.78
Staff physical activity behaviors	17.08 ± 2.78	12.51 ± 6.61	4.58 ± 6.16	0.07	0.74
Active opportunities	13.13 ± 3.95	12.5 ± 1.7	0.63 ± 5.79	0.77	0.11
Sedentary opportunities	18.33 ± 3.09	17.92 ± 4.69	.42 ± 6.28	0.86	0.07
Sedentary environment	13.33 ± 3.56	11.67 ± 5.90	1.67 ± 3.08	0.17	0.54
Portable play equipment	10.71 ± 4.52	11.43 ± 4.04	0.72 ± 3.97	0.63	0.18
Fixed play equipment	8.66 ± 3.4	9.54 ± 3.05	0.88 ± 2.51	0.36	0.35

^*a*^ Values presented are mean and standard deviation; differences between pre and post assessed using a paired t-test. *p < .05

## Discussion

Utilizing a community-based participatory research (CBRP) approach, the primary objective of this study was to determine the impact of a two-year intervention on ECE center food and PA policies, practices and environments. Throughout intervention development, implementation, interpretation and dissemination our intention was to utilize best-practices for engaging community stakeholders while at the same time paying particular attention to our specific context, allowing for flexibility to incorporate community knowledge and evidence and to adapt based on existing capacities of stakeholders involved. Results suggest that a tailored, participatory intervention has the potential to improve ECE center healthy eating and PA policies. Both written nutrition and PA policy significantly improved (*p* < 0.05), with greater improvements in PA policy (10.0 point increase, *p* = .048) as compared to nutrition policy (3.3 point increase, *p* = 0.02).

Similar improvements in written nutrition and PA policies have been achieved through traditional research interventions designed to equip ECE providers in evidence-based strategies to improve nutrition and PA policies and practices in diverse early childcare settings [19, 21–23, 25–27]. For example, in a seven-month randomized control trial in 17 ECE centers serving predominantly low-income families in California, Connecticut and North Carolina, Alkon et al. [27] demonstrated center-level improvements in both nutrition and PA policies using the NAP SACC intervention delivered by nurse child health consultants. Other studies, focused on either nutrition or PA policies, have also shown improvements. For example, Hollar et al. [23] observed significant improvements in written nutrition policies and nutrition training and education following nutrition training and technical assistance provided to ECE directors, faculty and staff serving racially and ethnically diverse, low-income children in Florida (FL). Similarly, LaRowe et al. [25] demonstrated improvements in PA policies and PA training and education following a one-year PA policy intervention in Wisconsin.

While several previous intervention studies have targeted ECE settings, the current study differs in important ways. While participatory research models, whereby academic and community partners are actively engaged throughout the research process, are key to the national prevention research agenda [44], few studies in the ECE setting have employed the CBPR methods utilized in the current study. Appel et al. conducted a two-year childhood obesity prevention pilot study in ECE settings that utilized CBPR methods, however results have not yet been published [45].

Importantly, participatory approaches empower community solutions and allow for adaptation to local needs. An important example of community-driven solutions is the incorporation of intensive individual-level TA tailored to the needs of each center (requested by ECE center directors) combined with group-level TA that facilitated networking and social support among participants (requested by local and state-level ECE stakeholders). An important example of adaptation is that while we initially had planned a 12-month intervention, feedback from stakeholders and study participants motivated the ECCAC to extend our study to 24-months. We learned that it took time for ECE directors and staff to buy-in to policy change. In our experience, the first 12 months were an important time for developing trust and achieving small wins. A recent Cochrane review identified 21 studies testing strategies to improve implementation of healthy eating and physical activity policies, practices or programs within ECE settings. Of the 21 identified, only 4 were longer than 12 months [46]. In contrast to the studies reviewed, the LWG ECE Initiative was community-initiated and implemented. While we partnered with a community coalition who provided intensive TA, the dose was still lower than is often tested in research studies who employ full intervention staff. We believe that our experience suggests that as communities consider how best to help centers implement standards, they need to consider the scope and extent of TA that is realistic and feasible. Building on the lessons learned from the pilot intervention and the trust developed between ECCAC partners, the LWG ECE initiative has now expanded to over 30 ECE centers across eight counties in Upstate South Carolina. Key features of the expanded initiative include: 1) a partner organization has assumed full responsibility for individual level TA with participating ECE centers, 2) the ECCAC is focused exclusively on group-based networking with participating ECE centers, and sharing lessons learned on how to intervene sustainably and how to utilize local resources, and 3) invited ECE centers serve low-income children and were recruited by coalition partners from a list of licensed childcare centers participating in the Child and Adult Care Food Program (CACFP).

There are several limitations that should be considered when interpreting the findings from our study. As a pilot study, we utilized a quasi-experimental study design that lacked a control group, and participating ECE centers were not randomly selected. As such, we cannot account for whether changes observed over the course of the two-year intervention were attributable to our intervention or to other concurrent ECE regulations or public health efforts, and generalizability of findings is limited. While our intervention was designed using the socioecological framework, we only collected data at the policy and environmental levels and, therefore, cannot assess whether policy changes resulted in individual level behavior changes. Additionally, observation dates were pre-arranged with centers and it is possible that provider and staff awareness of assessments biased results favorably. Because of the small sample size and short period of data collection, we did not measure inter-rater reliability and therefore it is possible that data collection staff experienced drift. Furthermore, our pilot study was not powered to detect statistically significant changes. Given the small sample size and exploratory nature of our pilot study, we did not adjust for multiple comparisons and readers should consider the possibility of Type I error in our findings.

## Conclusions

Our findings suggest that healthy eating and PA policy and environment change interventions that deliver high quality TA and utilize CBPR methods may help to promote healthy eating and active living opportunities for young children in ECE settings.

The CBPR approach was a critical component in the success of our pilot intervention. Personal relationships with LWG and ECCAC stakeholders were key in the recruitment of sites and the maintenance of their engagement throughout the two-year intervention process. As similar interventions are scaled to reach more ECE centers, CBPR components such as group size and selection of stakeholders need to be carefully considered.

## Data Availability

The datasets generated during this study are not publicly available due to the pilot nature of this small study. Additionally, permission for sharing this dataset or making it publicly available was not requested as part of the application for the Institutional Review Board; and hence, participant consent for inclusion of their data in such a dataset was not requested.

## Abbreviations

CBPR: Community-based participatory research
ECE: Early childhood education
ECCAC: Early childhood Community Advisory Committee
EPAO: Environment and Policy Assessment and Observation
LWG: LiveWell Greenville
PA: Physical activity
SC: South Carolina
TA: Technical assistance
